# Feasibility of thermocoagulation in a screen-and-treat approach for the treatment of cervical precancerous lesions in sub-Saharan Africa

**DOI:** 10.1186/s12905-016-0355-x

**Published:** 2017-01-07

**Authors:** Manuela Viviano, Bruno Kenfack, Rosa Catarino, Eveline Tincho, Liliane Temogne, Anne-Caroline Benski, Pierre-Marie Tebeu, Ulrike Meyer-Hamme, Pierre Vassilakos, Patrick Petignat

**Affiliations:** 1 Division of Gynaecology, Department of Gynaecology and Obstetrics, Geneva University Hospitals, Geneva, Switzerland; 2 Department of Biomedical Sciences, University of Dschang, Dschang, Cameroon; 3 Saint Damien Medical Centre, Ambanja, Madagascar; 4 Department of Gynecology and Obstetrics, University Centre Hospital, Yaoundé, Cameroon; 5 Geneva Foundation for Medical Education and Research, Geneva, Switzerland; 6 Division of Gynecology, Geneva University Hospitals, Boulevard de la Cluse 30, 1205 Geneva, Switzerland

**Keywords:** Human papillomavirus (HPV), Cervical intra-epithelial neoplasia (CIN), Cervical cancer, Screen-and-treat, Thermocoagulation

## Abstract

**Background:**

The use of thermocoagulation for the treatment of cervical precancerous lesions has recently generated a great deal of interest. Our aim was to determine the feasibility of this outpatient procedure in the context of a cervical cancer (CC) screen-and-treat campaign in sub-Saharan Africa.

**Methods:**

Between July and December 2015, women living in the area of Dschang (Cameroon) aged between 30 and 49 years, were enrolled in a CC screening study.

HPV self-sampling was performed as a primary screening test and women who were either “HPV 16/18/45-positive” or “positive to other HPV types and to VIA” were considered screen-positive, thus requiring further management. The primary outcome was the percentage of screen-positive patients who met the criteria to undergo thermocoagulation. The secondary outcome was the assessment of the procedure’s side effects immediately after treatment and at the 1-month follow-up visit.

**Results:**

A total of 1012 women were recruited in the study period. Among 121 screen-positive women, 110 of them (90.9%) were eligible to be treated with thermocoagulation. No patients discontinued treatment because of pain or other side effects. The mean ± SD (Standard Deviation) score measured on the 10-point Visual Analogue Scale (VAS) was 3.0 ± 1.6. Women having less than 2 children were more likely to report a higher pain score than those with more than two (4.2 ± 2.0 *versus* 2.9 ± 1.5, respectively; *p* value = 0.016). A total of 109/110 (99.1%) patients came to the 1-month follow-up visit. Vaginal discharge was reported in 108/109 (99.1%) patients throughout the month following treatment. Three patients (2.8%) developed vaginal infection requiring local antibiotics. No hospitalizations were required.

**Conclusion:**

The majority of screen-positive women met the criteria and could be treated by thermocoagulation. The procedure was associated to minor side effects and is overall feasible in the context of a CC screen-and-treat campaign in sub-Saharan Africa.

**Trial registration:**

The trial was retrospectively registered on November 11, 2015 with the identifier: ISRCTN99459678.

## Précis

Thermocoagulation is a feasible approach for the treatment cervical precancerous lesions in the low-resource context of sub-Saharan Africa.

## Background

Cervical cancer (CC) represents the fourth most common cancer among women worldwide [1]. Nevertheless, the global distribution of CC’s burden is marked by a significant disparity, with over 80% of cases occurring in low and medium-income countries (LMIC) [1]. Such disproportion is likely due to the difficulties in the implementation of a cytology-based screening program, which requires multiple-day, long-distance visits and important human and financial resources [2]. The availability of Point-of-Care technologies and visual inspection methods is progressively changing our understanding of CC screening and giving way to the possibility of offering screening and, if needed, treatment in a 1-day session [3, 4]. This same-day screen-and-treat strategy has proven to be more effective than cytology-based screening in terms of both patient compliance and costs [5]. In order to increase the screening strategy’s effectiveness, the choice of an appropriate treatment, which should be well accepted by women while guaranteeing satisfactory cure rates, is fundamental.

Excisional procedures such as Loop Electrosurgical Excision Procedure (LEEP) require trained personnel and expensive infrastructures, which makes them scarcely applicable in LMIC. Two low-cost and simple treatment methods are represented by cryotherapy and thermocoagulation. Cryotherapy is a highly effective intervention with a good cure rate, but the low availability of refrigerant gas makes its use challenging in LMIC [6]. In the given context, thermocoagulation may represent an attractive alternative for the treatment of cervical precancerous lesions.

Thermocoagulation has been available for several decades in Western countries. A recent meta-analysis including studies conducted mainly in industrialized countries has shown that the use of thermocoagulation for the treatment of CIN is as effective as other methods, such as cryotherapy and LEEP, with the advantage of being rapid and associated to a low occurrence of side effects [7]. This method may be of particular interest in the context of LMIC although to date, despite the growing interest and focus on this type of therapy, very little has been published about its use in developing countries.

An issue of particular interest is to determine, in the context of a screening campaign conducted in a low-resource setting, the percentage of screen-positive patients who meet the criteria to undergo thermocoagulation, as well as the side effects that can disrupt the procedure. These data would enable us to plan the allocation of resources in order to embody the procedure in a resource-constrained setting.

Our aim was to conduct a pragmatic analysis evaluating the feasibility of thermocoagulation in the context of a CC screen-and-treat campaign in sub-Saharan Africa.

## Methods

### Study setting

The Faculty of Medicine and Biomedical Sciences, Yaoundé, the National Committee for fight against cancer, Cameroon, the University of Dschang, Cameroon and the Geneva University Hospitals currently work together to evaluate innovative cervical cancer screening options in order to develop a screening approach adapted to the needs and means of people in Cameroon. This prospective study is part of this collaborative platform, which has been approved by the National Ethics Committee of Cameroon (2015/02/559/CE/CNERSH/SP) and by the Ethical Cantonal Board of Geneva, Switzerland (CCER 15–068). The study has been conducted at the District Hospital of Dschang, Cameroon. All participants were asked to sign a written informed consent form before taking part to the study. Assistance for data management was provided by the CRC (*Centre de Recherche Clinique*) of the Geneva University Hospitals.

### Study participants and procedure

Women aged between 30 and 49 years, interested to participate in a CC screening campaign were thoroughly informed about cervical cancer (basic facts, prevention, detection, treatment) and the study’s procedure. Exclusion criteria were pregnancy and previous total hysterectomy. The health care providers instructed all enrolled women on how to perform HPV self-sampling using a dry cotton swab (TCSSwabs, Technical Service Consultants Ltd, Lancashire, UK). The participants were told to gently unscrew the cap of the plastic tube in order to remove the dry swab. They then had to insert the soft-tip end of the swab into the vagina while being careful to avoid contact with external genitalia. When resistance was met (at about 5–6 cm), they had to gently turn the swab from three to five times clockwise and counterclockwise. Subsequently, the swab had to be placed back into the plastic tube. The procedure was performed by the women themselves.

The cervico-vaginal samples collected by the women were promptly analyzed with the HPV GeneXpert machine (GeneXpert®IV. Cepheid, 2015. Sunnyvale, California, USA). To do this, the swabs were first introduced in a NaCl 0,9% solution and vortexed for 3x15 s. Then, 1 mL of each sample was transferred to the cartridge and ran on the four-module GeneXpert machine. The GeneXpert HPV analysis consists of a real-time polymerase-chain-reaction (PCR) which uses, as an internal assay control for specimen adequacy, the detection of a Human reference gene (HMBS [hydroxymethylbilane synthase]) and an internal Probe Check Control (PCC). The PCC verifies reagent rehydration, PCR tube filling in the cartridge, probe integrity, and dye stability. This test includes reagents that allow the simultaneous detection of 14 hrHPV types (HPV16, 18, 31, 33, 35, 39, 45, 51, 52, 56, 58, 59, 66 and 68). The assay uses multiple fluorescent channels in order to detect individual types of HPV, groups of HPV, and the human reference gene. When sufficient signal is detected by the human reference gene, the assay results are reported as overall “positive”. In addition, if HPV16 and pooled HPV 18/45, together with other high-risk types are detected by the assay, the result will be labeled as either “positive” or “negative”. The HPV test results were available in less than an hour and the test was done on site, enabling us to screen, triage and treat women on the same day. The use of GeneXpert as a front-line cervical cancer screening test has been validated in a recently published study, which concluded that its performance and reproducibility are comparable to those of well-established HPV assays [8].

After obtaining the results of the sample analysis, HPV-negative women were advised to repeat screening after 5 years. HPV-positive women were invited to undergo visual inspection with acetic acid (VIA) as a triage test. Visual Inspection with Lugol’s Iodine (VILI) was also performed in order to aid delineate the pathological areas. VIA and VILI were performed by three trained local gynecologists. VIA consists in the application of a 4% acetic acid solution on the cervix, which may cause a slight burning sensation for a few minutes. The appearance of aceto-white areas touching the squamo-columnar junction (SCJ) helps define the pathological areas of the cervix. VILI consists in the application of Lugol’s iodine on the cervix, which does not cause any discomfort. The appearance of a well-defined, bright yellow area touching the SCJ defines the presence of a suspicious lesion. The application and interpretation of VIA and VILI were conducted according to the World Health Organization’s recommendations.

Women who were either “HPV-16/18/45-positive” or “positive to HPV-other high-risk types (HR-HPV) and to VIA” were considered as screen-positive, thus requiring treatment. Screen-positive women were considered eligible for thermocoagulation if (i) the lesion extended itself less than 2 mm in the endocervical canal, (ii) the anatomy of the cervix allowed to treat the entire lesion (multiple applications were possible), (iii) the lesion did not extend onto the vaginal wall, and (iv) if there was no suspicion of invasive cancer. No anesthetic prior to the procedure was administered. Treatment was performed using the thermocoagulator (WISAP® Medical Technology GmbH, Brunnthal/Hofolding, Germany) after delineation of the lesion using Lugol’s iodine. Treatment was performed with a probe heated to 100 °C, which was applied on the cervix for a period of 60 s. If necessary, the application was repeated in order to treat the entire abnormal area. After usage, the probe was washed with cold water, dried and heated for about 45 s at 120 °C to sterilize it. A cervicography of the cervix was undertaken before and after treatment for quality control.

### Outcome measures and follow-up

The primary outcome was the proportion of screen-positive women who were eligible for thermocoagulation. At the end of the procedure, the physician had to report whether any complications had taken place, as well as whether treatment had been completed specifying, if not, the reason why it had to be interrupted.

The secondary outcomes were the procedure’s side effects. The discomfort felt throughout thermocoagulation was assessed immediately after treatment by asking participants to report if they had felt pain, and to specify its intensity through the visual analogue scale (VAS). They had to indicate a point on a 10-cm line, with 0 meaning the procedure was not painful at all and 10 meaning it was the most painful experience one could imagine.

**Fig. 1:**
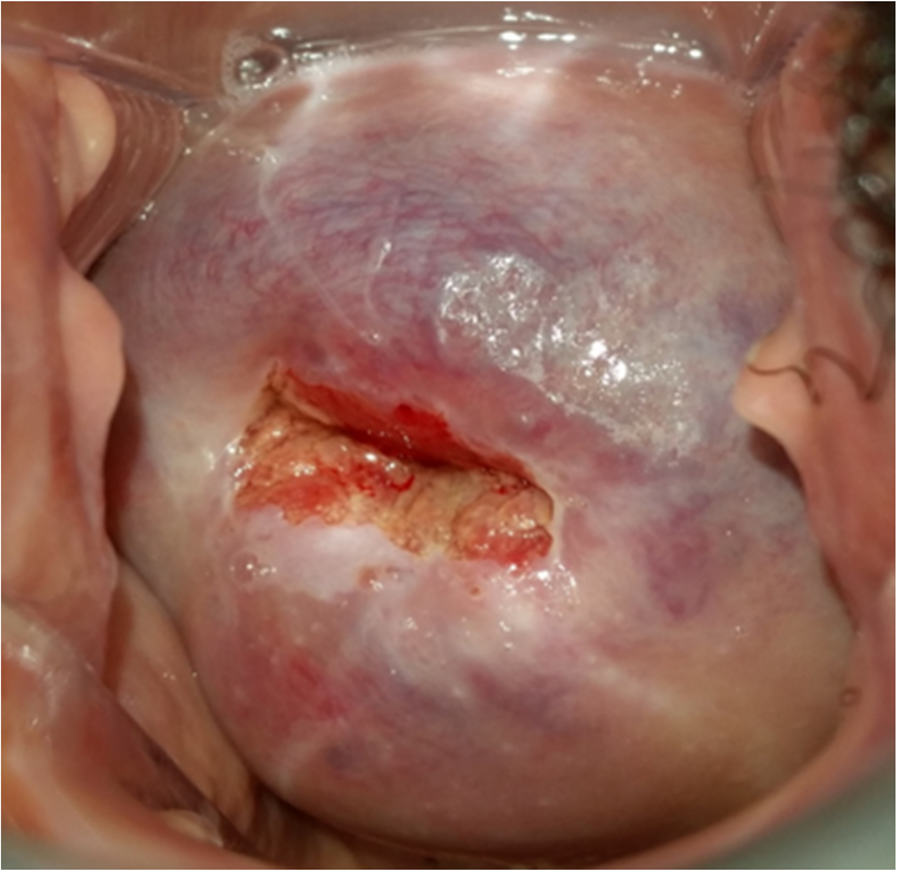
Picture of the native cervix

All treated women were invited for a follow-up visit at 1 month. The women’s subjective assessment of symptoms and side effects, as well as their duration in time, were registered. A vaginal examination was performed on all participants in order to exclude any infection and/or wound healing problem. Cervicography was also undertaken throughout this visit. Figure 1 shows the photographs taken on the native cervix, right after thermocoagulation treatment, and at the 1-month control visit.

### Data analysis and statistics

Data were analyzed with a statistical analysis software package StataCorp. 2013 (Stata Statistical Software: Release 13, College Station, TX, USA).

Quantitative variables were expressed as means and standard deviations, and qualitative variables were expressed as percentages, unless otherwise stated. Descriptive analyses were carried out in order to compare women by their socio-demographic characteristics, reproductive and sexual history, disease status and other aspects. Categorical variables were analyzed by Pearson’s chi-square or Fisher’s test and continuous and ordinal variables with the Mann-Whitney *U*-test or the Kruskal-Wallis test, when appropriate. All hypotheses were two-sided and *p*-values <0.05 were considered as statistically significant.

## Results

### Participants’ characteristics

A total of 1012 women underwent primary screening through HPV self-sampling. Sixty-one (6.0%, 95%CI:4.7–7.7) participants among those who underwent screening were positive for HPV-16/18/45, and were thus referred directly for treatment, while 126 (12.4%, 95%CI:10.6–14.6) women were positive for other HR-HPV types. Two of these women did not follow through with triage by VIA and VILI, corresponding to a dropout of 1.6% (95%CI:0.1–6.0). Among the 124 women who underwent triage with VIA, 60 (48.4%, 95%CI:39.8–57.1) of them were positive to these tests and were thus referred for treatment. Overall, 121 out of 187 HPV-positive women (64.7%, 95%CI:57.6–71.2) had a positive screen and were referred to further management. The characteristics of all screened women are reported in Table 1.

**Table 1:** Socio-demographical characteristics and obstetric and gynecological h istory of the screened population

Variable	*N*		%
Total number of women screened	1012		
Age (mean ± SD), y		39.6 ± 5.6	
Age groups, y			
30–34	232		22.9
35–39	257		25.5
40–44	280		27.7
≥ 45	241		23.9
Marital Status			
Without a partner	55		5.5
With a partner	954		94.6
Education			
Unschooled	7		0.7
Primary education	223		22.1
Secondary education	618		61.3
Tertiary education	157		15.6
Work			
Employee/Independent/Farmer	720		71.1
Housewife	259		25.6
Other	31		3.1
Age at menarche (mean ± SD), y		14.8 ± 1.9	
Age of first sexual intercourse (mean ± SD), y		18.0 ± 2.8	
Number of sexual partners (mean ± SD)		3.7 ± 2.7	
Number of pregnancies, (mean ± SD)		5.5 ± 2.3	
Number of children (mean ± SD)		4.5 ± 1.9	
Age at first delivery (mean ± SD), y		21.8 ± 4.0	
Contraception			
Pill/Injectable/Intrauterine device/Implanon/Other	186		18.4
Condom	111		11.0
None	709		70.5
Antecedents of cytological screening			
Yes	222		78.0
No	788		22.0
HPV type			
HPV 16	18		9.6
HPV 18/45	27		15.5
Other HR-HPV	124		66.8
HPV-16 and other HR-HPV	2		1.1
HPV-18/45 and other HR-HPV	13		7.0
VIA/VILI status among other HR-HPV positive women			
Pathological	60		48.4
Non pathological	64		51.6

*Abbreviations*: *SD* standard deviation, *y* years, *HPV* human papillomavirus

### Primary outcome-percentage of screen-positive women who met cold coagulation criteria

Overall, 110 out of 121 (90.9%, 95%CI:84.3–95.0) screen-positive women were considered eligible for thermocoagulation and treated. No patients discontinued treatment because of pain or other adverse effects. A total of 8 (7.3%, 95%CI:3.5–13.9) patients were ineligible for thermocoagulation. Ineligibility reasons included inability to identify the cervix, no visualization of the SCJ or the presence of a lesion going more than 2 mm inside the cervix (*n* = 5), and having a lesion suspicion of cancer (*n* = 3). Three (2.7%) patients were excluded from the study. These include 2 women not identified as pregnant at the time of enrolment due to an inclusion error and one who could not be treated because of a technical issue due to impossibility of heating the probe. Patients with large pathological areas or having a lesion suspicious for cancer were referred to LEEP or hysterectomy. Pregnant patients were invited for a follow-up visit after childbirth.

### Secondary outcomes—procedure’s side effects

The participants were asked to quantify the discomfort felt throughout thermocoagulation using the VAS. A mean ± standard deviation (SD) pain score of 3.0 ± 1.6 was reported. Thermocoagulation never had to be interrupted because of pain. Table 2 reports the results of the thermocoagulation assessment immediately after treatment.

**Table 2:** Thermocoagulation assessment immediately after treatment and at the 1-month control visit

	Variable	*N*		%
Immediately after treatment	Total women treated	110		90.9
	Pain			
	Yes	105		95.5
	No	3		2.7
	Not specified	2		1.8
	VAS pain score (mean ± SD)		3.0 ± 1.6	
	VAS pain score (median, range)		2 (1–8)	
	Desire of future pregnancy	54		49.1
At the 1-month control visit	Total women seen at the 1-month visit	109		99.1
	Pain			
	Yes	34		31.2
	No	75		68.8
	VAS pain score (mean ± SD)		0.8 ± 1.4	
	Pain duration (mean ± SD), days		2.1 ± 4.8	
	Vaginal discharge			
	Yes	108		99.1
	No	1		0.9
	Vaginal discharge duration (mean ± SD), days		16.2 ± 8.4	
	Cicatrisation			
	Yes	100		91.7
	No	9		8.3

### Adverse events

No serious adverse events occurred during the procedure or in the 30 days following treatment. Three patients (2.8%) consulted prior to the 1-month control visit because of important vaginal discharge, and a vaginal antibiotic therapy was prescribed to them.

### Impact of socio-demographics and obstetric and gynecological history on pain assessment

Table 3 reports the association of pain according to socio-demographics and past obstetric and gynecological history. The patient’s age did not have a significant impact on pain perception. Women with 2 or more children were more likely to feel less pain compared to those who had only one or no children (2.9 ± 1.5 vs 4.2 ± 2.0, *p* = 0.016).

**Table 3:** VAS score according to socio-d emographics and obstetric and gynaecological history

Variable	Mean ± SD	*p* value
Age groups, y		0.5
30–34	3.0 ± 1.7	
35–39	3.3 ± 1.6	
40–44	2.8 ± 1.7	
≥ 45	2.8 ± 1.4	
Marital Status		0.08
Without a partner	3.7 ± 1.3	
With a partner	3.0 ± 1.6	
Education		0.844
Unschooled	0	
Primary education	2.8 ± 1.2	
Secondary education	3.1 ± 1.7	
Tertiary education	3.0 ± 1.7	
Work		0.84
Employee	3.2 ± 1.8	
Independent	3.1 ± 1.8	
Housewife	2.8 ± 1.4	
Farmer	2.4 ± 0.8	
Other	3.4 ± 1.7	
Number of sexual partners		0.411
0–4	3.1 ± 1.6	
≥ 5	2.9 ± 1.6	
Number of children		0.016
0–1	4.2 ± 2.0	
≥ 2	2.9 ± 1.5	

*Abbreviations*: *SD* standard deviation, *y* years

### Follow-up at 1 month

The 1-month follow-up was achieved for 109 out of 110 treated patients, corresponding to a drop out of 0.9% (95%CI:<0.0001–5.5). Overall, 34 (31.2%, 95%CI:23.2–40.4) patients reported having felt some degree of pain, mainly during the first few days following treatment, while 75 (68.8%, 95%CI:59.6–76.8) women did not experience any. The mean ± SD pain intensity was 0.8 ± 1.4, with a mean ± SD duration of 2.0 ± 4.2 days. Vaginal discharge, sometimes with little blood, was reported in 108 out of 109 (99.1%, 95%CI:94.5–100.0) patients, with a mean ± SD duration of 16.2 ± 8.4 days. Adequate wound healing was reported in 100 out of 109 (91.7%, 95%CI:84.9–95.8) women; the remaining ones showed delayed cervical healing and were prescribed a local antibiotic. These results are also reported in Table 2.

**Fig. 2:**
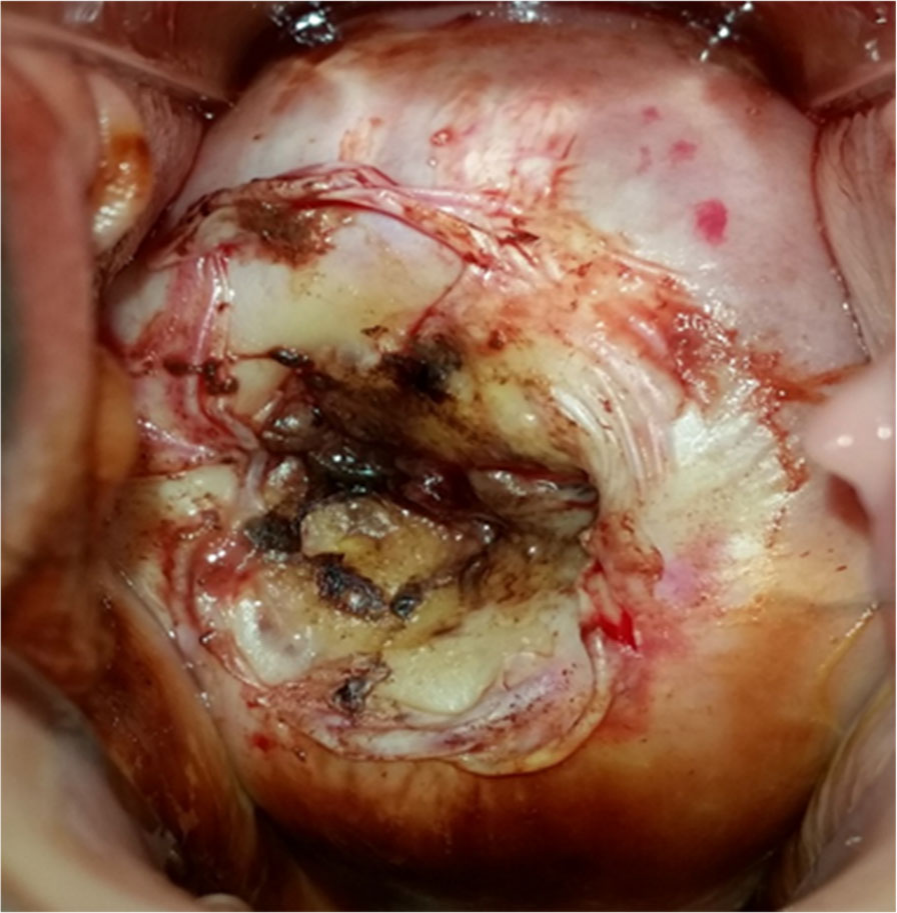
Picture of the cervix right after treatment with thermocoagulation

**Fig. 3:**
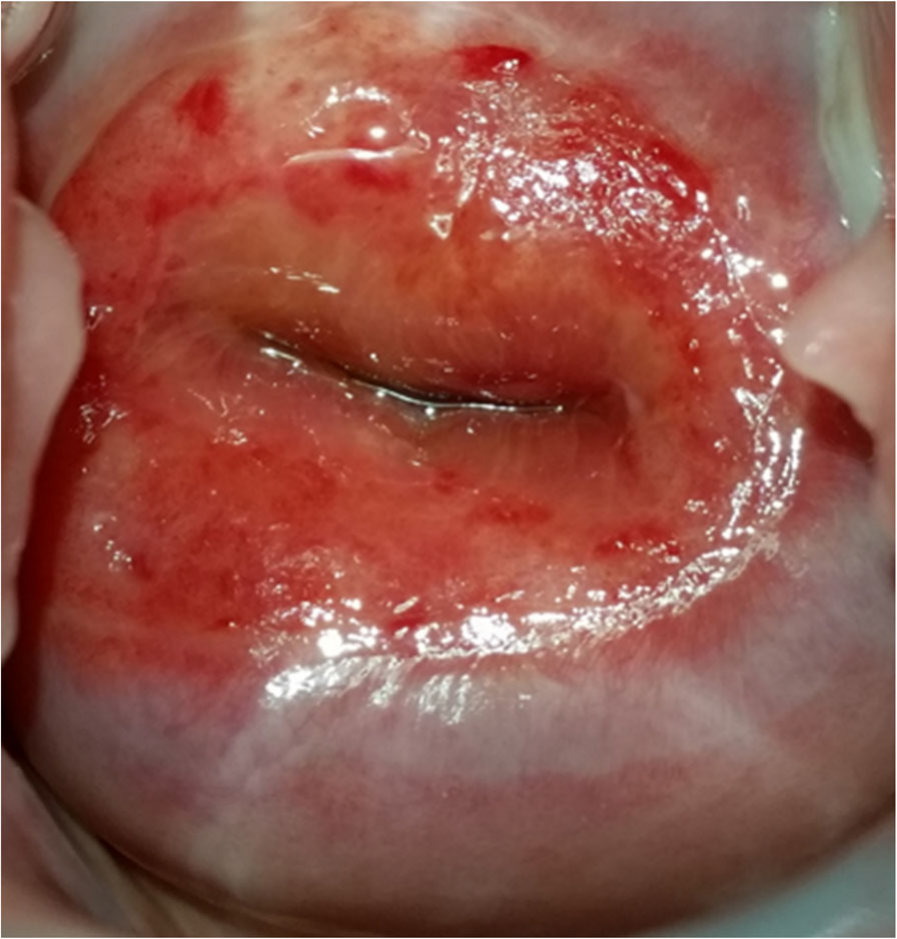
Picture of the cervix at the 1-month control visit following thermocoagulation

Figures 1, 2 and 3 show sequential pictures of the cervix before, immediately after treatment, and as at the 1-month follow-up visit, respectively.

## Discussion

In a sample of more than 1000 women participating in an HPV-based cervical cancer screening campaign, most screen-positive participants met the criteria to undergo thermocoagulation in the context of a screen-and-treat approach, with less than 10% of them not fulfilling the eligibility criteria and needing to be referred for LEEP or a more radical type of CC treatment. These results are comparable to those obtained in a VIA-based cervical cancer screening campaign involving 6 African countries and almost 20,000 screened women, where 87.7% of VIA-positive women were considered eligible for cryotherapy [9]. Another study conducted in Peru found that 83.8% of women screened as positive upon HPV testing and triage by VIA were suitable candidates for cryotherapy [10].

According to our experience, thermocoagulation is a quick and easy procedure to learn and perform in the African context. The thermocoagulator costs approximately the same as a small cryosurgical unit [11]. When compared to cryotherapy, its potential advantages in clinical practice include (i) the possibility to precisely destruct the abnormal area under direct visual control, (ii) the possibility to treat large abnormal areas in their entirety with several applications of the probe, and (iii) the fact that it requires a shorter treatment time than cryotherapy. Finally, like cryotherapy, it does not require anesthesia [12].

Our triage algorithm allowed all HPV-16/18/45-positive women, who have a higher risk of CIN2+ when compared to women positive for other HR-HPV genotypes to be promptly treated, whereas women positive for other HR-HPV types were only treated in presence of pathological VIA/VILI [13]. This type of management was applied in an effort to reduce the rate of overtreatment by selecting, among HPV-positive women, those who have a higher risk of presenting CIN [14].

No patients in our study discontinued treatment because of pain or other adverse effects, thus demonstrating that thermocoagulation is a low-pain procedure that can be successfully carried out until the end if patients are correctly informed and sufficiently cooperative [15]. Women generally expressed minimal discomfort, and those who experienced pain described it essentially as lower abdominal cramping, which was felt mainly during the procedure and immediately after it. As the main side effects associated to the procedure, such as bleeding and vaginal discharge, mostly occur between 1 and 6 weeks following thermocoagulation, all patients were asked to come for a control visit at 1 month following treatment [16, 17]. At this follow-up visit, almost all treated women reported having experienced a watery vaginal discharge, seldom with little blood, for about 2 weeks, while only a few of them experienced abdominal pain. No complications requiring hospitalization, such as pelvic inflammatory disease or other issues, took place.

Strengths of this study are represented by the fact that it describes the main key points associated with this quite unexplored technique in the context of sub-Saharan Africa. In addition, we have assessed the eligibility or participants for thermocoagulation and the overall feasibility of this technique in the context of a CC screen-and-treat program. Finally, we have a minimal loss to follow-up at 1-month, which allows us to validate the low complication rate in the 30 days that follow treatment. Limitations that need to be addressed are the short follow-up period and the absence of results regarding the cure rate.

This is one of the largest studies conducted in LMIC on the use of thermocoagulation for the treatment of cervical precancerous lesions. The aim of this pragmatic and descriptive analysis is to improve knowledge about a procedure that is potentially widely applicable in LMIC for the management of screen-positive women. Further prospective studies should focus on evaluating the long-term efficacy of thermocoagulation in preventing the development of CIN and CC, as well as its impact on fertility in LMIC. In our series, about half of the women still wished to become pregnant in the future, and the health care provider has to take this aspect into consideration. To date, there aren’t any known adverse effects of thermocoagulation on fertility, but this issue still needs to be investigated with long-term follow-up studies [7].

## Conclusions

The high availability of thermocoagulation, together with its efficacy, renders it a valuable option for the treatment of screen-positive women in LMIC, such as those in sub-Saharan Africa [11, 18, 19]. The simplicity of this intervention may encourage the shift of this type of treatment from hospital-based environments to community settings, as the device weights approximately 2 kg and is thus easy to move and, globally, ideal for all remote areas that dispose of electricity. Further research should focus on the achievable cure rates and its impact on fertility in order to determine if thermocoagulation, rather than cryotherapy, may become the new gold standard for treatment of screen-positive women in the sub-Saharan context.

## Abbreviations

CC: Cervical cancer
CIN: Cervical intra-epithelial neoplasia
HPV: Human Papillomavirus
HR-HPV: High-risk Human Papillomavirus
LMIC: Low- and middle-income countries
SD: Standard deviation
VAS: Visual analogue scale
VIA: Visual inspection with acetic acid
VILI: Visual inspection with Lugol’s Iodine
Y: Years
